# Does Deep Cervical Flexor Muscle Training Affect Pain Pressure Thresholds of Myofascial Trigger Points in Patients with Chronic Neck Pain? A Prospective Randomized Controlled Trial

**DOI:** 10.1155/2016/6480826

**Published:** 2016-11-21

**Authors:** Pavlos Bobos, Evdokia Billis, Dimitra-Tania Papanikolaou, Constantinos Koutsojannis, Joy C. MacDermid

**Affiliations:** ^1^Roth McFarlane Hand and Upper Limb Centre Clinical Research Laboratories, St. Joseph's Health Centre, Western University, London, ON, Canada; ^2^Department of Physical Therapy, Technological Educational Institute of Western Greece, Patras, Greece

## Abstract

*Background.* We need to understand more about how DNF performs in different contexts and whether it affects the pain threshold over myofascial trigger points (MTrPs).* Purpose.* The objectives were to investigate the effect of neck muscles training on disability and pain and on pain threshold over MTrPs in people with chronic neck pain.* Methods.* Patients with chronic neck pain were eligible for participation with a Neck Disability Index (NDI) score of over 5/50 and having at least one MTrP on either levator scapulae, upper trapezoid, or splenius capitis muscle. Patients were randomly assigned into either DNF training, superficial neck muscle exercise, or advice group. Generalized linear model (GLM) was used to detect differences in treatment groups over time.* Results.* Out of 67 participants, 60 (47 females, mean age: 39.45 ± 12.67) completed the study. Neck disability and neck pain were improved over time between and within groups (*p* < 0.05). However, no differences were found within and between the therapeutic groups (*p* < 0.05) in the tested muscles' PPTs and in cervicothoracic angle over a 7-week period.* Conclusion.* All three groups improved over time. This infers that the pain pathways involved in the neck pain relief are not those involved in pain threshold.

## 1. Introduction

Pain in the cervical region is a pathological condition which is associated with increasing disability in the general population [1–3]. The process of cervical pain is best described as episodes with various degrees of recovery that can happen over a lifetime [4]. The persistent deterioration of neuromuscular control of the neck muscles partly contributes to chronicity and recurrence of the neck problem as reported in previous studies [5]. One of the neuromuscular compromises for neck pain has been shown to be the low or delayed activation on the deep neck flexors (DNFs) muscles (particularly longus capitis and longus colli) [6]. Thus, conservative treatments often prescribed from physicians include both (deep and superficial) muscle training strategies [7, 8]. Therapeutic exercising programs that are focused on the training of cervical muscles have been shown to reduce pain and disability with promising results [9, 10]. More specifically, clinical trials that included either DNF training or superficial muscle training have demonstrated to effectively reduce chronic neck pain and disability [9, 10].

Myofascial trigger points (MTrPs) are known factors that can be associated with neck pain [11]. It has been suggested that MTrPs are neck pain-generating sources for mechanical pain. However, few studies have included treatment for MTrPs for the management of this type of pain [1, 12]. Referred pain depends on the sensitivity of MTrPs and, therefore, active MTrPs may play a pivotal role in the conveyance of pain in more general painful conditions [12].

Although deep and superficial cervical flexors training have shown to reduce pain and disability, it is unknown whether deep or superficial cervical muscles are more effective in improving neck disability and pain. Also, it is not clear whether exercise training of these sets of muscles can have an effect on the MTrPs of patients with neck pain. Therefore, given the above, the first objective was to determine the effect of neck muscle training on patients' disability and pain in the cervical region. The second objective was to investigate the effect on pain pressure thresholds (PPTs) over MTrPs in chronic neck pain patients.

## 2. Methods

### 2.1. Trial Design

This was a prospective single-blind randomized controlled trial with 3 therapeutic groups in Patras, Greece, with trial registration number ISRCTN13364486. The first group was the deep neck flexor group (Group A), the second was the superficial muscle (Group B), and the third was the advice group (control). Ethical approval was granted from the scientific committee of the Department of Physiotherapy of the Technological Educational Institute of Western Greece.

### 2.2. Participants

Patients between 18 and 65 years old with idiopathic chronic neck pain were invited to participate in the study. The selection of the sample was done through notifications and advertisements in local hospitals, rehabilitation centres, and social meeting places across the greater area (Achaia, Greece). Patients eligible for participation must have had neck pain for at least 3 months (chronic), disability score 5/50 in Greek version of the Neck Disability Index (NDI) [13], and at least one active or latent MTrP in any of the muscles: levator scapulae, upper trapezoid, and splenius capitis. Patients were excluded from participation if they had history of previous neck surgery, cervical radiculopathy, any systemic disease, myopathy, pregnancy, and pathological conditions of the central nervous system or if they had participated in a physiotherapy training program in the last 6 months.

### 2.3. Procedure

Subjects who met the eligibility criteria completed the patient reported outcomes (PROs) and an assessment form which included information about the study, consent form, personal information, a brief medical history, demographic features, and a pain body diagram. The measurements and the training programs were performed by 2 physiotherapists who were trained in the intervention protocol to improve consistency of treatment between providers. Prior to the start of the study pilot studies were used to improve the consistency of the clinical measurement. After that, patients were examined for the presence of MTrPs in the following muscles: upper trapezoid, levator scapulae, and splenius capitis and the pain pressure thresholds were measured. In the end of this phase, all patients were digitally photographed and they performed the craniocervical flexion test (CCFT). The training program of the DNF group (A) was based on the CCFT level that they achieved in their first assessment and it was their starting point. All participants received instruction guidelines through written leaflet and digital video disk (duration of 17 minutes recorded by the two physiotherapists) with ergonomic pieces of advice and exercise guidelines for neck for a fuller understanding of the execution of the therapeutic exercises. All therapeutic interventions (for the two exercise groups described below) were provided twice per week, for approximately 30–40 minutes per session for 7 consecutive weeks. The difficulty of exercises was gradually increased (intensity and repetitions) depending on the response demonstrated by each patient. All the measurements (self-reported outcomes and clinician based outcomes) were performed at the beginning and at the end of the therapeutic training programs. Also, patients were instructed not to participate in other therapeutic sessions and/or receive any medication during the study.

### 2.4. Interventions

All the individuals were provided with an exercise leaflet guide and a digital video disk on how to perform the reported exercises at home. The program was divided into 3 parts, the warm-up, the basic part, and the ergonomic guidelines with stretching part. In the warm-up patients were instructed to do the following: slow rotations of the head in all directions, movement of the shoulders in all directions, and rotation of the arms in all directions. Patients were instructed to breathe normally during the warm-up period. In the basic part patients were instructed to do posterior movement of the neck from sitting position, posterior movement of the neck with towel or elastic belt from sitting position, isometric contraction of neck muscles in all directions (flex-extension, side flexion-extension, and rotation left–right and forward and backward of the neck), and by adding resistance with their hand very slow rotary motion of the head. In the stretching part, patients were instructed to do neck extension-flexion stretching, neck side flexion, and rotation stretching. In the end all the groups performed streching exercises. The group A and B performed the stretching part with the assistance of physiotherapists. The group C had written instructional guidelines and a digital video disk on how to perform the stretching part alone in their house. Ergonomic directions were given and patients were repeatedly instructed in the optimal position of how to sleep, drive, and sit on a chair.

### 2.5. Deep Neck Flexors' Training Group

The DNF group (A) performed the following exercises: the craniocervical flexion test (CCFT) with air pressure biofeedback [6], nodding from supine position, nodding from pronation position, and nodding from sitting position close to the wall [14]. All exercises were terminated if the patient activated the superficial neck muscles.

### 2.6. Superficial Muscle Group

The superficial muscle group (B) performed posterior head movement from sitting position with elastic band, posterior head movement from supine position, movement in all directions from pronation position, and “cat-camel motion exercise” [15].

### 2.7. Control Group

The advice group (C) performed only the home exercise program described in the leaflet and the digital video disk.

### 2.8. Outcomes

The patient reported outcome (PRO) measures were the Neck Disability Index (NDI) [16], which was the primary outcome measure, the numeric pain rating scale (NPRS) [17] of pain intensity and the Client Satisfaction Questionnaire-8 (CSQ-8) [18], for measuring patient satisfaction, and short form-12 (SF-12) [19] for health status. The psychometric properties of the Greek version of NDI have been assessed by Trouli et al. [13] and found to be reliable, valid, and responsive tool. The NPRS has been shown to be reliable and responsive tool in patients with mechanical neck pain [20]. The Greek version of SF-12 has been found to be a reliable, valid, and responsive tool [21]. The clinician based outcome (CBO) measures were the pain pressure thresholds measured with a mechanical algometer and the craniocervical flexion test (CCFT) with air pressure biofeedback unit REF and the cervicothoracic angle with digital imaging [22].

#### 2.8.1. Pressure Pain Threshold (PPT) Measurements

The PPTs over active or latent Myofascial Trigger points (MTrPs) of levator scapula, upper trapezoid, and splenius capitis muscles (both sides) were tested with Wagner pressure pain algometer (FDK-20 model). MTrPs were clinically palpated according to Travell and Simons diagnostic criteria in order to verify the reproducibility of the location [23]. The PPTs were measured on each muscle for both sides and also one reference point of the PPT was measured in the xiphoid process for checking the degree of perception of pain. The examiner advised the participants to indicate (a) the first sign of pain and (b) the point where pain could not be tolerated. The PPT measurement yielded excellent intra- and intertester reliability, tested in 15 healthy subjects during 2 (or 3) consecutive days in the pilot (ICC between 0.83 and 0.89).

#### 2.8.2. Craniocervical Flexion Test (CCFT)

The CCFT measured the DNF muscle endurance with Chattanooga pressure biofeedback unit. The application of the craniocervical flexion test followed the protocol that was described by Jull at al. [24]. The examiners instructed the patients how to perform the craniocervical flexion movement before the test and included one practice trial. The test process involved 5 increments of continuous difficulty [24]. The intraexaminer reliability was tested in 15 healthy subjects at the same time during two consecutive days and it was very high (intraexaminer reliability ICC = 0.91).

#### 2.8.3. Cervicothoracic Angle

The measurement of cervicothoracic angle is a clinical measurement [22] that indicates the neck posture and is defined as the angle between C7 spinal process, the middle of the ear tragus, and the horizontal level. The cervicothoracic angle measures the angle produced between the horizontal plane of the picture and the line of C7 spinal process and the tragus of the ear for the traction of the head. The digital photo pictures were taken with a Nikon Coolpix P520 and the analyses of the angles were done with digital protractor in Photoshop software. The reduced value of this angle is an indication for forward head posture [25]. For this clinical measurement, a digital camera Nikon Coolpix P520 was placed in a stable base in the kinesiotherapy lab of the institution, 3 metres away from each patient calibrated in a specific place for the whole duration of the study. The participant position for measurement was standardised supported sitting and participants were asked to stare at a stable marker placed in the wall, ahead of them. Reliability of the cervicothoracic angle was measured in 15 healthy subjects with digital protractor with Photoshop CS3 software (3 consecutive days) and yielded excellent intraexaminer reliability ICC = 0.91.

### 2.9. Randomization

Participants were randomly assigned by the leader investigator into one of the three therapeutic groups without knowing their therapeutic group, utilising computer-based randomization software. Randomization took place on the day they signed the informed consent and each therapist from each group was informed.

### 2.10. Statistical Analysis and Sample Size

For the data analysis descriptive statistics were used to establish the clinical and demographic features of our sample. The dependent variables analyzed were the NDI (primary outcome measure), the NPRS, SF-12, the cervicothoracic angle, the CCFT, and the PPTs of the MTrPs. A repeated measures' generalized linear model was used to determine time and treatment group effects controlling for age and gender as covariates. The Client Satisfaction Questionnaire-8 (CSQ-8) was analyzed by using a one-way analysis of variance following by a post hoc Tukey test. The significance level was set at *α* = 0.05, 95% CI.

An a priori sample size calculation was performed with anticipated effect size for the primary outcome measure based on mean differences of change between groups [20] (Cohen's *d*: 0.8, desired statistical power level: 0.8, and probability level: 0.05). The minimum sample size per group was 21 (one-tailed hypothesis). A power analysis was performed to calculate the power of the sample size (60 subjects) with power strength 0.80 and as primary outcome measure the Neck Disability Index [16] based on mean differences of change between groups [20]. The observed power was 0.92 (one-tailed hypothesis).

## 3. Results

**Figure 1:**
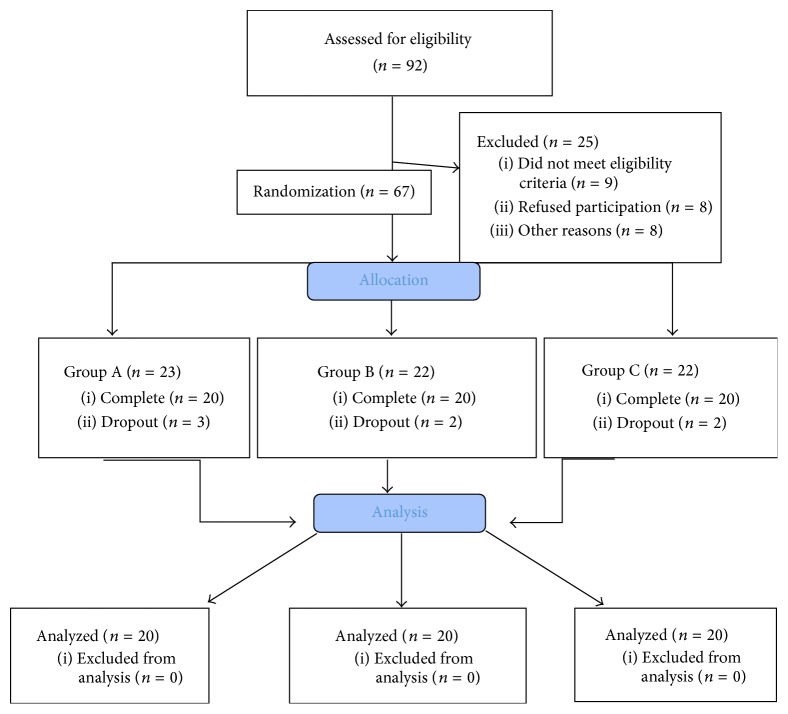
Flow diagram.

**Figure 2:**
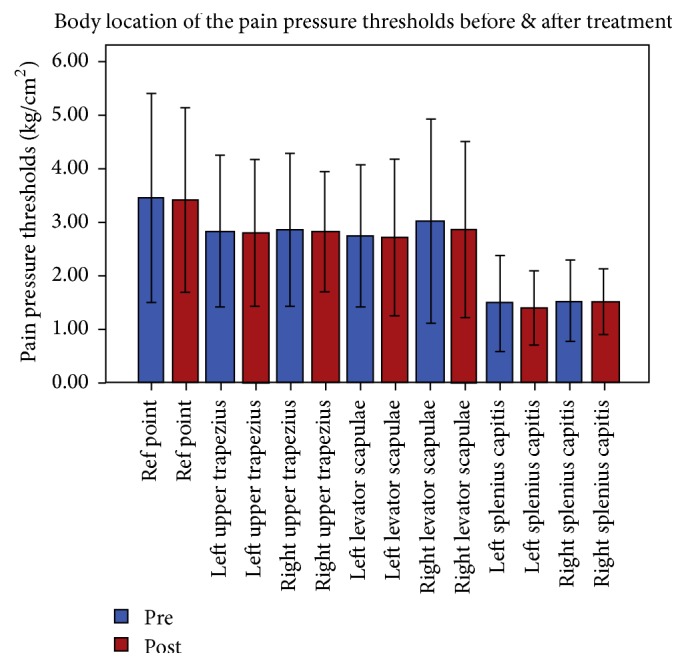
Sensitivity of pain pressure thresholds on both sides before and after treatment.

**Figure 3:**
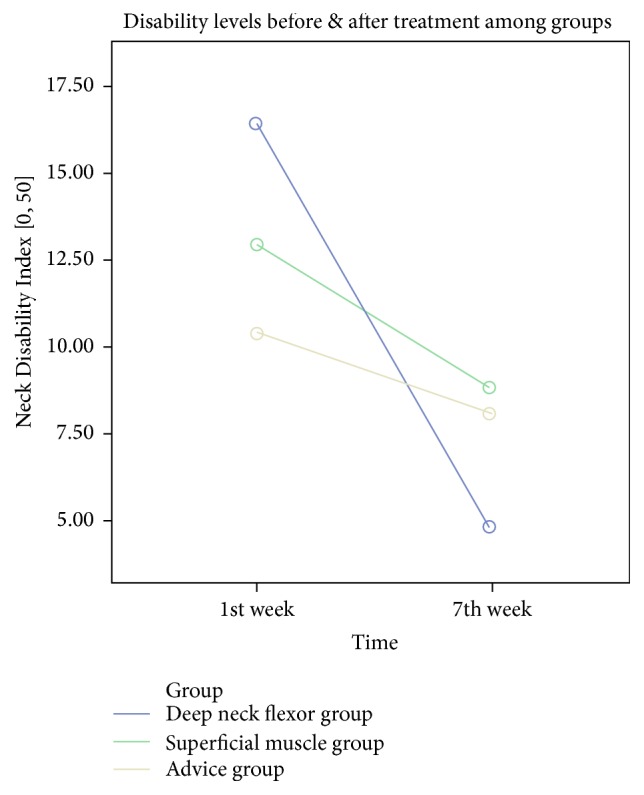
Measurement of disability levels among groups before and after treatment.

Out of the 92 initially approached patients, 83 patients met the eligibility criteria, and 67 agreed to participate in the study and were, thus, randomized. Following allocation, 7 more patients dropped out before the end of the trial; thus, 60 (12 males and 48 females, mean age 39.45 ± 12.67) completed the study (Figure 1). The participants were randomized into Group A: 2 males and 18 females, mean age: 38.45 ± 12.67, Group B: 5 males and 15 females, mean age: 40.45 ± 13.47, and Group C: 6 males and 14 females, age: 39.45 ± 13.46. The sample's demographic information is summarized in Table 1. In total, 360 muscles were assessed for the presence of MTrPs where 304 muscles had active MTrPs and 26 latent MTrPs and 30 muscles did not have any MTrPs. Pain was measured on 3 different levels in the NPRS (“pain now,” “pain at best,” and “pain at worst”) in all groups. The generalized linear model (GLM) revealed that there is a statistically significant difference in all pain subscales (Table 2) over time for the deep neck flexor group (A) and the superficial neck muscle group (B). However, in the advice group (C) the pain intensity levels had statistically significant differences and decreased over time in two out of three pain intensity levels (NPRS “pain now” and “at worst”) while the NPRS “pain at best” had no statistically significant difference in the end of the training program. Disability was measured with NDI which was the primary outcome measure and the GLM analysis revealed that in all interventions groups disability levels were reduced over time (Figure 3). In terms of health-related quality of life there were no statistically significant differences regarding physical and mental levels. The craniocervical flexion test (CCFT) demonstrated statistically significant improvement over time only for the deep neck flexor group compared to the other 2 treatment groups. The statistical analysis for the cervicothoracic angle found no statistically significant difference among the three therapeutic groups. The pain pressure thresholds were measured in both sides of the cervical muscles being tested and the smallest values of sensitivity were recorded on splenius capitis (Figure 2). Minimal change was observed after treatment at any of the MTrPs. The splenius capitis had a lower pain threshold compared to the trapezius and levator scapulae muscles. The statistical analysis revealed that the sensitivity of pain pressure thresholds (Table 3) had no statistically significant difference across the groups over time in the end of the therapeutic interventions.

**Figure 4:**
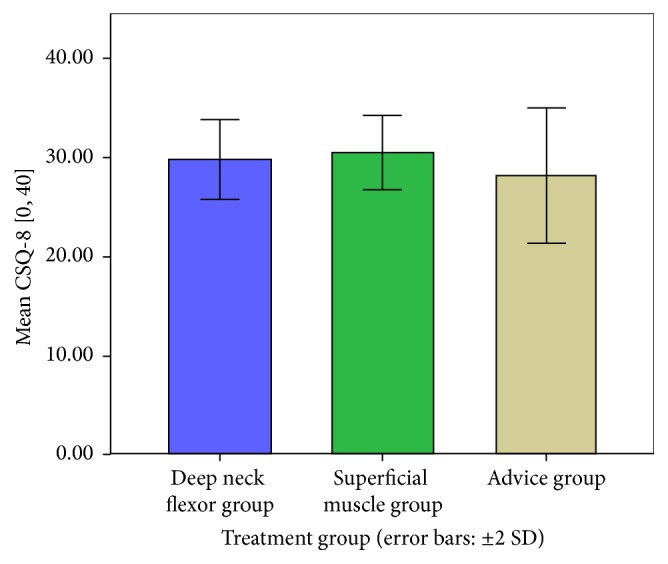
Patients satisfaction levels (CSQ-8) among treatment groups in the end of the intervention.

The one-way analysis of variance of the patients' satisfaction (CSQ-8) revealed that the superficial muscle group (B) had greater satisfaction compared to the third group (control) but overall all groups had very high satisfaction levels (Figure 4).

## 4. Discussion

The results of this study showed that specific progressive training programs that are targeting the deep and superficial muscles are capable of reducing the pain and disability. However, they are not capable of changing the sensitivity of the PPT over MTrPs on the specific extensor and upper back muscles and the head posture. The descriptive analysis of the age of the sample in each group showed that there was homogeneity in the patients' age. Furthermore, the high prevalence of women compared to men in each group in terms of neck pain condition is clearly reflected in this clinical study confirming the available international literature [4].

Regarding neck pain intensity, the results of this study confirm the positive effects of the DNF exercise in reducing pain and disability levels. Previous randomized trials, wherein the therapeutic interventions aimed at improving the activation of DNFs, have shown that they are effective in patients with chronic neck pain, since by increasing the activation of those muscles the pain and disability were decreased [6, 26, 27]. In particular, the education of DNF in the intervention group (A), after application of progressive exercise program to muscles for seven weeks, was able to reduce all 3 categories of sample pain levels on NPRS, showing a statistically highly significant difference compared with initial pain scores. Also, the superficial neck muscle group (B) equally decreased the NPRS pain levels, on charges of NPRS “pain at best” to have decreased slightly less than the intervention group (A). Pain levels decreased similarly to the control group (C) but only on two pain categories of the NPRS (present and worst pain intensity), while not observing any statistically significant difference in pain levels of the category of NPRS “pain at best” before and after treatment. More generally, therapeutic training groups B and C, which aimed to exercise the neck superficial muscles of the cervical spine lasting 7 weeks, showed that Groups B and C were able to reduce pain levels, a fact confirmed by the very recent literature [28, 29].

The neck disability levels for the treatment groups (A, B, and C) were found statistically significant (*p* < 0.001); however the control group was found with markedly smaller decrease compared with the other two groups. These results show that a progressive exercise program of the deep neck flexors and superficial muscles lasting 7 weeks is sufficient to reduce the disability and neck pain. In contrast, the reduction was inferior to the control group, which may be explained by the fact that the patients did not have the supervision and guidance of the clinician (they only had an audio visual and manual material). This observation maybe highlights the importance of the presence of the physical therapist during treatment sessions.

Regarding the measurements of health-related quality of life with SF-12 no change was observed due to the fact that all the initial measurements among groups were within the normal range. This was an unexpected finding since chronic conditions tend to have lower Physical and Mental Composite Scores (PCS and MCS) [21].

Although therapeutic intervention individually in each group reduced the levels of disability and pain, however, there was no effect on the sensitivity of the pressure limits of MTrPs right and left in the levator scapula, the splenius capitis, and upper trapezius (*p* > 0.05) after treatment in each group. The initial values of the PPT of MTrPs in the levator scapula and upper trapezius showed different values between groups and between the right and left side within the same group. Instead, the original values of PPT in splenius capitis did not differ among themselves or between groups or between the right and left side because the ranges of the values measured in this muscle were very small (Figure 2). Generally, the majority of previous research studies found that the PPT levels on the cervical extensor muscles are not affected by the application activity in these muscles [30–32]. In contrast, the study of Sharan et al. [29], a sample of eight people (physiotherapists), showed great differences (statistically significant) between the initial and final measurements of PPT levels after applying yoga-term program 4 weeks. However, the absence of a control group and insufficient explanation and presentation reliability of the material and the methodology used by Sharan et al. [29] and the omission of the cervical muscles in which the PPT were measured make the study vulnerable to potential biases.

The additional clinical measurements carried out, such as cervicothoracic angle, revealed that no group had difference before and after treatment. This suggests that the forward head posture may not be related to pain or disability in the neck or that a progressive therapeutic intervention program of DNFs and exercise of the neck superficial muscles for seven weeks is not able to significantly change the head posture. Previous studies that compared the head posture before and after treatment after training program in healthy subjects revealed contradictory findings, which maybe can be attributed to the different methodological techniques on the measurement of head posture and the large heterogeneity of age of the sample [33].

Regarding neck endurance, the craniocervical flexion test (CCFT) of the deep neck flexors group reached the maximum level of CCFT which was obvious because it was the main exercise for the therapeutic group (A). However, the other two groups were capable of reducing disability and pain but in the end the CCFT was not statistically significant before and after treatment. This can be explained by the fact that their exercises were more focused on the superficial muscles compared to the deep neck flexors. In the current literature so far there are no other studies that compared the CCFT between the deep and superficial muscle flexors by controlling the pain and disability levels.

The strengths of this study are the comparison between deep and superficial neck flexors on neck pain and disability levels as well as the measurement of 360 MTrPs and the measurement of head posture. To our knowledge our study may indicate the importance of differential diagnosis regarding the management of neck pain.

## 5. Limitations

The randomization of the sample had some differences in terms of baseline levels of NDI and the NPRS category “pain at worst.” In particular, the disability indicator of NDI used as the primary evaluation tool indicated a heterogeneity between intervention group (A) and a control group (C) (*p* = 0.010), suggesting that the DNF group was more disabled. It can be seen that the DNF group had the highest disability among the other two and therefore it can be assumed that it had the greater reduction in disability. The 7 persons' “dropout” (following randomization) could have influenced the initial homogeneity values on NDI between groups. The absence of the blind effect of the examiners and the fact that the sample had a high prevalence of females (80%) are the limitations of our study. However, the fact that chronic neck ache incidence affects mostly women can partly justify this discrepancy across men and women. Therefore, males in our sample are underrepresented since all our dropouts were males. Also, a long term follow-up will probably have had a more significant outcome. Also, the fact that the therapy was performed from two different therapists indicates the possibility that maybe one therapist outperformed the other one.

## 6. Conclusions

All therapeutic groups showed adequate reduction in disability and pain but there was no effect on pain pressure thresholds over the sensitivity of MTrPs of splenius capitis, upper trapezoid, and levator scapulae across groups. No statistically significant changes were yielded on the PPTs of the cervical muscles being tested, possibly implying a different pain mechanism pathway (than specific or general exercise-induced training) for MTrPs relief. Moreover, the progressive training programs of the deep and superficial neck flexors muscles did not have any alteration on sample's cervicothoracic angle. Further research in this area would be of great value.

## Figures and Tables

**Table 1:** Sample demographic and clinical features.

Demographic features	*n*	%
*Gender*		
Female	47	80
Male	13	20
*Residency*		
Urban	14	23
Suburban	42	70
Rural	4	7
*Education*		
Lower education	2	3
Secondary education	5	8
Higher education	53	89
*Clinical features*		
*Tingling*		
Yes	5	8
No	55	92
*Weakness*		
Yes	3	12
No	57	88
*Numbness*		
Yes	3	5
No	57	95
*Onset of symptoms*		
Sudden	17	28
Gradually	43	72
*Duration of symptoms*		
3 months	3	5
6 months	9	15
12 months	19	32
24 months	16	27
48 months	13	21

**Table 2:** Mean change scores and significance level results.

Measurement	Deep neck flexor group (Α)	Superficial muscle group (Β)	Advice group (C)	Group effect (*p*)		
				A	B	C
NPRS, pain now	3.47	1.63	0.99	*p* < 0.001^†^	*p* < 0.001	*p* < 0.001
NPRS, pain at best	1.69	0.67	0.18	*p* < 0.001	*p* = 0.006	NS^‡^
NPRS, pain at worst	2.57	1.98	1.98	*p* < 0.001	*p* < 0.001	*p* < 0.001
Neck Disability Index	11.60	4.12	2.26	*p* < 0.001	*p* < 0.001	*p* = 0.004^*∗*^
CCFT	−6.66	−1.61	−0.92	*p* < 0.001	NS	NS
Cervicothoracic angle	1.29°	−5.79°	−1.00°	NS	NS	NS
SF-12, MCS	1.03	−5.63	−1.84	NS	NS	NS
SF-12, PCS	−7.94	−3.75	−3.45	NS	NS	NS

NPRS: numeric pain rating scale, CCFT: craniocervical flexion test, ^*∗*^statistically significant difference (*p* < 0.05), ^†^extremely statistical difference (*p* < 0.01), and ^‡^nonsignificance effect.

**Table 3:** Mean scores (kg/cm^2^) and SD with significance level results of pain pressure thresholds.

Tested muscles		Deep neck flexor group (Α)		Superficial muscle group (Β)		Control group (C)		Group^*∗*^ effect (*p*)			Time^*∗*^ sex (*p*)	Time^*∗*^age (*p*)
		Pre	Post	Pre	Post	Pre	Post	A	B	C		
UT	L	2,29 (0,62)	2,52 (0,57)	2,95 (0,86)	3,13 (1,01)	3,04 (0,47)	2,89 (0,63)	NS	NS	NS	*p* = 0.025^*∗*^	NS^‡^
	R	2,36 (0,57)	2,5 (0,42)	2,89 (0,87)	3,07 (0,94)	2,96 (0,44)	2,86 (0,56)	NS	NS	NS	NS	NS

LS	L	1,54 (1,22)	1,66 (1,2)	3,18 (1,14)	3,17 (1,18)	1,98 (1,28)	1,92 (1,24)	NS	NS	NS	NS	NS
	R	1,76 (1,34)	1,73 (1,27)	3,39 (1,34)	3,22 (1,20)	2,48 (1,08)	2,4 (1,07)	NS	NS	NS	NS	NS

SC	L	1,61 (0,59)	1,47 (0,49)	1,26 (0,67)	1,43 (0,58)	1,39 (0,27)	1,35 (0,28)	NS	NS	NS	NS	NS
	R	1,45 (0,63)	1,41 (0,56)	1,37 (0,56)	1,57 (0,35)	1,5 (0,26)	1,39 (0,16)	NS	NS	NS	NS	NS

UT: upper trapezius, LS: levator scapula, SC: splenius capitis, ^*∗*^statistically significant difference (*p* < 0.05), ^†^extremely statistical difference (*p* < 0.001), and ^‡^nonsignificance.
